# Supplementary material for: Evaluation of cortical thickness and cortical thickness index in the proximal femur—CT, dual energy absorptiometry (DXA), trabecular bone score (TBS) –
Source: PLoS One. 2025 Feb 27;20(2):e0312420. doi: 10.1371/journal.pone.0312420 (PMC11867387; doi:10.1371/journal.pone.0312420)
Supplement: Attachment — Submitted filename: Response to Reviewers.docx [file pone.0312420.s001.docx]

**Respond to academic editor**

1. Please ensure that your manuscript meets PLOS ONE's style requirements, including those for file naming

**=> We changed Scipts style considering on PLOS manual style templit**

**: Followed Title , Author , Affiliation formating guideline etc.**

2. At this time, please address the following queries

“The authors received no specific funding for this work”

**=> we included this comment on cover letter**

3. Thank you for stating the following in your Competing Interests section:  
"NO authors have competing interests"

**=> “The authors have declared that no competing interests exist. “ was included on cover letter**

4. Data availability

**=> We uploaded our patient data on figshare and it’s DOI is 10.6084/m9.figshare.24755277**

5. Please upload a new copy of Figure 1 as the detail is not clear.

**=> Figure 1 ‘s resolution has been changed and upload a new copy**

6. Please include a caption for figure 1.

**=> Added caption for figure 1**

7. Please include captions for your Supporting Information files at the end of your manuscript, and update any in-text citations to match accordingly.

**=> We changed all caption for supporting information and uploaded end of our manuscripts. it also match accordingly**

**Additional respond to academic editor**

Methods:
• The study design is a cross sectional study made from patient records and not from previous cross sectional study. Correct the study design

**=> We modified the study design to fit**
• It’s better to write the methods section into subtitles, it was written in non sectional unclear different ideas like “ study population with inclusion and exclusion criteria and study sitting, data collection, statistical analysis, …………………

**=> The method section was modified with subtitles and statistical analysis was moved to the method section.**

• Stsastical analysis: is a section under methods sections. The details of analysis are deficient

=> **statistical analysis was moved to the method section. and supplemented the details.**

Results: All tis “
• All titles of the tables are deficient and some are wrong as “ Table 3:. The statistical significance in two groups “

=> **all table’s title was changed and made them fit**

• The tests of significance were not mentioned as footnotes

**=> The statement related to the table was written on footnote.**

• Correct the significance from 0.000 to <0.001

**=> We fixed it**
• No correlation test between measurements was done

• Multivariante analysis was not done
**=> It was difficult to conduct correlation tests and multivariant analysis studies because there were many missing information due to insufficient samples. However, this study is a meaningful paper in analyzing the correlation between cortical bone thickness and fractures in Far East Asia. It is unfortunate that the above research was omitted, but it was difficult due to the limitations of sample data.**

- Discussion:
• No strengths and limitations were included
• No recommendations for further studies

**=> We added limation and recommendation for futher studies**

**Respond to reviewer**

Reviewer #1: The manuscript provides the cortical thickness and and cortical thickness index from CT scan, T-score and TBS data based on DXA in total of 600 patients from South Korea. These data source is valuable in 3D printing. The findings on correlation between fracture and bone health are consistent with the literature.

The statistical analysis section mentioned all parameters were compared by Student's t-test. It works for the comparing two groups (Table 2,3,4). However, the Table 5 and table 6 listed 6 age groups, please specify which statistical tests were used for calculating p-value.

**=> Not all were done with study t-test. The statistics of tables 5 and 6 you mentioned were modified to use ANOVA because there was a mistake when writing it.**

Table 1 present the average data of proximal femoral geometry from CT. Authors need to specify what are the numbers follow the average, are they range of the mean i.e. (min ~max)?

**=> I wrote the part you told me ( min ~ max ) on footnote.**

Also, considering the different body size between male and female, it is necessary to provide the average data by sex. Fourth, the patients included were heterogenous. A more proper and accurate geometry of the proximal femur should be obtained from the homogenous group, such as the whole non-fracture group.

**=> As a retrospective study conducted with a limited sample, it was difficult to perform the classification according to the gender you mentioned. If research based on other samples is conducted in the future, it is believed that academic achievement will be achieved through meta-analysis.**

Reviewer #2: The work presents a retrospective analysis of the proximal femur that can bring important clinical information, however the analysis of some parameters can improve the work.
The purpose of the mentioned study should be changed in order to encompass all the analysis carried out. It seems to me that changing to: Characterizing the proximal femur of patients without alterations, with fractures...in different parameters...would be more appropriate.

**=> What we wanted to show was to study the correlation between fractures and anatomical features, including femoral proximal cortical bone thickness. This required a comparison between fracture and non-fracture patients. There were relatively many data on fracture patients in hospitals, so it was possible to measure and compare the opposite side, but in the case of non-fracture patients, it was difficult to perform CT and other tests for the necessary data**

**The non-facture group was formed by collecting data from bone necrosis patients of the femoral head or suspected fracture patients who came to the outpatient clinic, and due to the nature of this medical institution, sufficient data was not accumulated, so it is regrettable that gender comparisons were not made. However, the purpose of the study was to correlate the fracture with the anatomical values of the proximal femur.**

How did the sample of 200+200+200 arrive, was it by chance? Explain the methodology in detail.
**=> We changed the method section to However, among the patients who visited our hospital without fractures, it was very difficult to find patients who performed all the necessary tests when we studied. Therefore, among patients without fractures, 200 patients who had completed the examination were collected and compared with patients with fractures.**

Show pictures of examples of trochanteric fracture and femoral neck fracture.
**=> We attached the femur neck fracture as figure 2 and the trochanetic fracture as figure 3, and the original figures 2,3,4,5,6,7 were revised to 4,5,6,7,8,9 respectively.**

Tables must be self-explanatory and in the title mention the type of sample used

**=> We have added a new detailed description of the table to the footnote at the bottom of the table**

Statistically, age and gender should also be studied in the different parameters analyzed, using only the sample of 200 normal patients as a reference. Therefore, a different and more complete statistical analysis is recommended.

**=> It was difficult to conduct different and more complete statistical analysis studies because there were many missing information due to insufficient samples. However, this study is a meaningful paper in analyzing the correlation between cortical bone thickness and fractures in Far East Asia. It is unfortunate that the above research was omitted, but it was difficult due to the limitations of sample data.**
