# Supplementary material for: Evaluation of cortical thickness and cortical thickness index in the proximal femur—CT, dual energy absorptiometry (DXA), trabecular bone score (TBS) –
Source: PLoS One. 2025 Feb 27;20(2):e0312420. doi: 10.1371/journal.pone.0312420 (PMC11867387; doi:10.1371/journal.pone.0312420)
Supplement: Attachment — Submitted filename: Respond to reviewer.docx [file pone.0312420.s002.docx]

Respond to reviewer

Reviewer #1: In the Data collection section, author stated “propensity score matching was performed to compare the data of the two groups” (i.e. hip fracture group and non-fracture group). Suggest authors specifying this selection method in the table 2 footnote and describing what parameters were included in the propensity score calculation.

**Thank you for your good question, we included only age and sex factors for the propensity score matching. We added comment about this in the manuscript.**

For Table 6, authors need to specify what are the numbers after the +/- sign.

**+/- sigh is standard deviation, we included explanation about this**

The presentation of N=600 row is not consistent across the 8 columns.

**Thank you for your sharp point, we corrected this at the table 6**

The last two columns showed mean (+/-) but not the other 6 columns. Authors shall fix the inconsistency

**Thank you for your detail comments, we added standard deviation to the other 6 columns**
